# Effects of 6-Month Sitagliptin Treatment on Metabolic Parameters in Diabetic Patients Taking Oral Glucocorticoids: A Retrospective Cohort Study

**DOI:** 10.14740/jocmr2153w

**Published:** 2015-04-08

**Authors:** Hisayuki Katsuyama, Akahito Sako, Hiroki Adachi, Hidetaka Hamasaki, Hidekatsu Yanai

**Affiliations:** a Department of Internal Medicine, National Center for Global Health and Medicine, Kohnodai Hospital, Chiba 272-8516, Japan; b Clinical Research Center, National Center for Global Health and Medicine, Kohnodai Hospital, Chiba 272-8516, Japan

**Keywords:** Body weight, Glucocorticoid, HbA1c, Sitagliptin

## Abstract

**Background:**

There are no guidelines for the treatment of diabetes in patients taking glucocorticoids. We studied to understand the effects of 6-month treatment with sitagliptin on metabolic parameters in diabetic patients taking glucocorticoids.

**Methods:**

We retrospectively picked up patients who had been prescribed sitagliptin for 6 months during the continuous prescription of oral glucocorticoids between October 2010 and October 2013 by a chart-based analysis, and compared the data before the sitagliptin treatment with the data at 6 months after the sitagliptin treatment started.

**Results:**

Fifteen patients were eligible for the analyses in our study. The plasma glucose and HbA1c levels were significantly reduced by the sitagliptin treatment. Furthermore, body weight significantly decreased. We found a significant and inverse correlation between the change in HbA1c levels and HbA1c levels at baseline. However, there was no significant correlation between the change in HbA1c levels and the daily glucocorticoid dose at baseline.

**Conclusions:**

The present study demonstrated that sitagliptin significantly reduced plasma glucose, HbA1c and body weight. Further, sitagliptin was more effective to improve glycemic control in patients taking glucocorticoids with higher HbA1c levels, independently of the daily glucocorticoid dose.

## Introduction

Glucocorticoids are widely prescribed anti-inflammatory and immunosuppressive drugs. Although glucocorticoids provide beneficial effects on inflammatory, allergic, immunologic and malignant diseases, glucocorticoids induce various adverse reactions including osteoporosis, hypertension and hyperglycemia [1-4]. Glucocorticoid-induced insulin resistance and pancreatic islet-cell dysfunction lead to mild increase in fasting plasma glucose levels and a greater increase in postprandial glucose levels [4-9]. The treatment for diabetes in patients taking glucocorticoids may be different from that in diabetic patients without taking glucocorticoids. Although there are no guidelines for the treatment of diabetes in patients taking glucocorticoids, short-acting prandial insulin therapy is currently recommended [10]. Several small studies showed the effectiveness of α-glucosidase inhibitors [11], thiazolidinediones [12, 13], and the glucagon-like peptide-1 (GLP-1) receptor agonist [14], for the treatment of diabetes in patients taking glucocorticoids; however, the conclusions led by these studies are premature.

Sitagliptin is one of the dipeptidyl peptidase-4 (DPP-4) inhibitors and is widely used in the treatment of type 2 diabetes. DPP-4 inhibitors enhance levels of active incretin hormones such as the GLP-1 and the glucose-dependent insulinotropic polypeptide (GIP) which are released from the intestinal cells following the meal ingestion [15]. The GLP-1 and GIP stimulate insulin secretion from pancreatic β cells and the GLP-1 inhibits glucagon secretion from pancreatic α cells, which reduces plasma glucose levels. These incretin-based therapies improve postprandial hyperglycemia and have low risk of hypoglycemia due to their glucose-dependent action [16, 17]. Therefore, DPP-4 inhibitors may be useful in the management of hyperglycemia in patients with glucocorticoids-induced diabetes, which is characterized by normal or mild increase in fasting plasma glucose levels and a remarkable increase in postprandial glucose levels. However, the evidence is lacking for the effects of DPP-4 inhibitors for these patients. Here, we retrospectively studied effects of 6-month treatment with sitagliptin on metabolic parameters in diabetic patients taking glucocorticoids.

## Materials and Methods

### Subjects

This study was approval by the Institutional Ethics Committee in National Center for Global Health and Medicine (NCGM-G-001603), and was also performed in accordance with the Declaration of Helsinki. This study was registered with the University Hospital Medical Information Network (UMIN) clinical trials registry, number UMIN 000014508.

We selected patients who had been prescribed sitagliptin for 6 months during the continuous prescription of oral glucocorticoids between October 2010 and October 2013 by a chart-based analysis. Patients with type 1 diabetes were excluded. We also excluded patients who had started taking sitagliptin before the initiation of glucocorticoid therapy, who had taken intravenous glucocorticoids during the study periods, and whose oral glucocorticoid prescription was discontinued during the study period.

### Methods

We compared the data before the sitagliptin treatment with the data at 6 months after the sitagliptin treatment started. Body weight, blood pressure, plasma glucose, HbA1c, serum low-density lipoprotein-cholesterol (LDL-C), triglyceride (TG), high-density lipoprotein-cholesterol (HDL-C), aspartate aminotransferase (AST), alanine aminotransferase (ALT) and γ-glutamyltransferase (γGTP) in studied subjects were measured almost at the same time point before and after 6-month treatment with sitagliptin. LDL-C values were determined by the direct measurement or calculated by using the Friedewald formula. All data are expressed as mean ± SD.

### Statistical analysis

Comparison of the variables determined before and after the sitagliptin treatment was analyzed by a paired Student’s *t*-test. Pearson’s simple correlations coefficients were performed to determine the correlations between the data before the start of sitagliptin treatment and changes in HbA1c after the sitagliptin treatment. P < 0.05 was considered to be statistically significant.

## Results

We found 892 patients who had taken sitagliptin between October 2010 and October 2013, and 114 patients had taken oral glucocorticoids at least once during this period. Fifteen patients who had taken sitagliptin for 6 months during the continuous prescription of oral glucocorticoids were eligible for the analyses in our study.

Table 1 shows the baseline characteristics and autoimmune, inflammatory and allergic diseases treated with glucocorticoids. Eleven subjects were diagnosed as glucocorticoid-induced diabetes and four subjects were diagnosed as type 2 diabetes exacerbated by glucocorticoids. The glucocorticoid dose was with the equivalent of 7.8 ± 6.0 mg/day of prednisolone at baseline, and 5.7 ± 4.2 mg/day after the sitagliptin treatment with no significant change (P = 0.191). The dose of sitagliptin was 47 ± 8 mg/dL at baseline and 55 ± 24 mg/dL after the sitagliptin treatment (P = 0.173). Other hypoglycemic agents taken before and after the sitagliptin treatment were shown in Table 2. Five subjects were treated with the sitagliptin monotherapy during the study period.

**Table 1:** Baseline Characteristics of Subjects Studied (n = 15)

Age	66.9 ± 13.9
Sex (M/F)	6/9
Body height (cm)	156 ± 11
Body weight (kg)	57.5 ± 11.4
Body mass index (BMI) (kg/m^2^)	23.5 ± 3.0
Diseases treated by glucocorticoids	
Interstitial pneumonia	2 (13%)
Primary multiple myositis	2 (13%)
Polymyalgia rheumatica	2 (13%)
Dermatomyositis	1 (7%)
Rheumatoid arthritis	1 (7%)
Adult-onset Still’s disease	1 (7%)
Eosinophilic granulomatosis with polyangitis	1 (7%)
Sarcoidosis	1 (7%)
Panhypopituitarism	1 (7%)
Bullous pemphigoid	1 (7%)
Chronic urticaria	1 (7%)
Bronchial asthma	1 (7%)

**Table 2:** Other Hypoglycemic Agents Which Subjects Had Taken Before and After the Treatment With Sitagliptin (n = 15)

Hypoglycemic agents	Pre-treatment	Post-treatment
None	6 (40%)	6 (40%)
Sulfonylureas	1 (7%)	4 (27%)
Biguanides	2 (13%)	4 (27%)
Thiazolidinediones	3 (20%)	3 (20%)
α-glucosidase inhibitors	5 (33%)	4 (27%)
Glinides	4 (27%)	0 (0%)
Insulin	1 (7%)	1 (7%)

Table 3 shows the changes in the clinical parameters after the 6-month sitagliptin treatment. The plasma glucose and HbA1c levels were significantly reduced by the sitagliptin treatment. Furthermore, the body weight significantly decreased.

**Table 3:** Changes in Variables After 6-Month Sitagliptin Treatment (n = 15)

	Subjects studied (n)	Pre-treatment	Post-treatment	P value
Body weight (kg)	9	61.6 (9.9)	58.9 (10.6)	0.001
Systolic blood pressure (mm Hg)	11	129 (18)	134 (17)	0.390
Diastolic blood pressure (mm Hg)	11	72 (10)	73 (16)	0.914
Plasma glucose (mg/dL)	15	195 (72)	121 (23)	0.004
HbA1c (%)	15	7.6 (0.9)	6.5 (0.9)	< 0.001
LDL-cholesterol (mg/dL)	9	107 (24)	100 (20)	0.079
Triglyceride (mg/dL)	11	133 (55)	163 (64)	0.228
HDL-cholesterol (mg/dL)	8	51 (16)	52 (14)	0.934
AST (IU/L)	14	30 (21)	26 (13)	0.508
ALT (IU/L)	14	32 (28)	24 (12)	0.302
γGTP (IU/L)	8	32 (12)	31 (21)	0.856
eGFR (mL/min/1.73m^2^)	14	86 (33)	80 (35)	0.029

**Figure 1:**
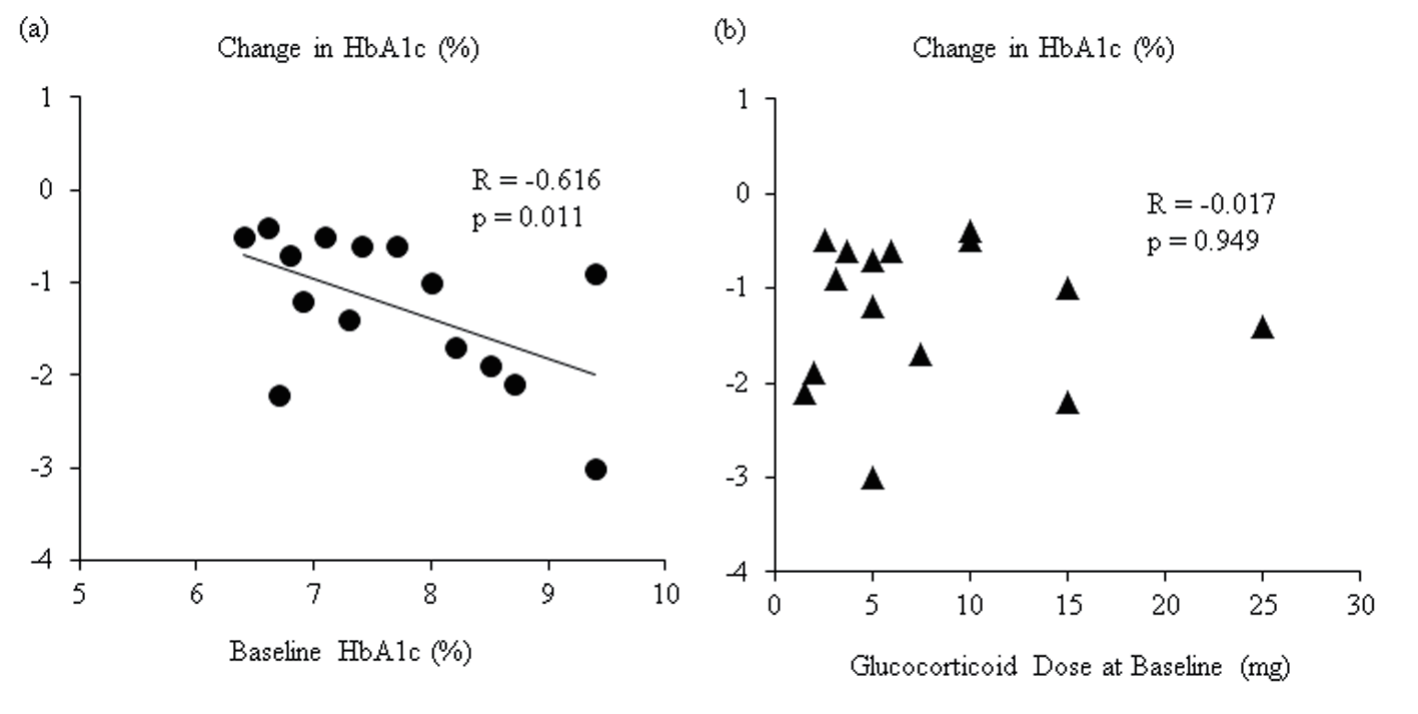
(a) Correlation between changes in HbA1c after 6-month sitagliptin treatment and HbA1c levels at baseline. (b) Correlation between changes in HbA1c after 6-month sitagliptin treatment and daily glucocorticoids dose at baseline.

We analyzed the correlation between change in HbA1c levels and HbA1c levels at baseline. We found a significant and inverse correlation between the change in HbA1c levels and HbA1c levels at baseline (Fig. 1a). However, there was no significant correlation between the change in HbA1c levels and the daily glucocorticoid dose at baseline (Fig. 1b).

Subgroup analyses were performed in five subjects (two males and three females) treated with the sitagliptin monotherapy to exclude the influence of other hypoglycemic agents. Four subjects were diagnosed as glucocorticoid-induced diabetes and one patient was diagnosed as having type 2 diabetes. The mean ± SD of age and BMI were 54.7 ± 12.8 years old and 23.5 ± 2.8 kg/m^2^, respectively. Table 4 shows the changes in the clinical and biochemical parameters after the 6-month sitagliptin monotherapy in these subjects. The HbA1c levels also significantly decreased after the sitagliptin treatment by 1.04%, and plasma glucose levels showed a tendency to decrease. Body weight also showed a tendency to decrease by 3.5 kg.

**Table 4:** Changes in Variables After 6-Month Sitagliptin Monotherapy (n = 5)

	Subjects studied (n)	Pre-treatment	Post-treatment	P value
Body weight (kg)	3	64.6 (3.6)	61.1 (2.5)	0.087
Systolic blood pressure (mm Hg)	4	126 (12)	133 (17)	0.568
Diastolic blood pressure (mm Hg)	4	75 (4)	83 (10)	0.345
Plasma glucose (mg/dL)	5	192 (76)	107 (22)	0.060
HbA1c (%)	5	7.0 (0.3)	5.9 (0.7)	0.036
LDL-cholesterol (mg/dL)	2	135 (4)	116 (18)	0.465
Triglyceride (mg/dL)	4	131 (44)	128 (36)	0.957
HDL-cholesterol (mg/dL)	3	59 (16)	48 (11)	0.148
AST (IU/L)	5	30 (16)	28 (19)	0.789
ALT (IU/L)	5	30 (17)	26 (14)	0.710
γGTP (IU/L)	3	39 (11)	35 (24)	0.789
eGFR (mL/min/1.73 m^2^)	5	111 (36)	108 (40)	0.486

## Discussion

The present study demonstrated that the sitagliptin treatment including the add-on therapy and monotherapy significantly improved HbA1c levels, indicating the effectiveness of sitagliptin for the treatment of diabetes in patients taking glucocorticoids. Glucocorticoids have been reported to show the dose-dependent effect on glucose metabolism [10]. However, our study showed that the sitagliptin treatment improved HbA1c levels, independently of the daily glucocorticoid dose, which also supported the usefulness of sitagliptin for the treatment of diabetes in patients taking glucocorticoids.

The underlying mechanisms for glucocorticoid-induced hyperglycemia include increased hepatic endogenous glucose production, reduced insulin-stimulated glucose uptake in skeletal muscle, increased visceral fat deposition and insulin resistance [4, 5, 18]. Moreover, glucocorticoids may impair insulin secretion from β cells and may augment glucagon secretion from α cells [4, 6, 7]. Since glucocorticoids usually cause mild increase in fasting plasma glucose and a greater increase in postprandial glucose, rapid-acting prandial insulin therapy without basal insulin is currently recommended [10]. However, insulin therapy increases the risk of adverse effects, such as hypoglycemia and weight gain. Furthermore, it is too difficult to determine the optimal insulin dose, because the dose of glucocorticoids is frequently changed due to the severity of the diseases treated by glucocorticoids.

Several small studies showed the effectiveness of oral hypoglycemic agents. Tanaka et al reported the treatment for steroid-induced diabetes with α-glucosidase inhibitor, voglibose (1 day). They treated six patients and showed a decrease in the amount of urinary glucose [11]. Willi et al evaluated the efficacy of a thiazolidinedione, 5 - 8 weeks troglitazone treatment, in seven patients with long-standing steroid-induced diabetes, and they found that troglitazone significantly reduced HbA1c from 7.8±0.4% to 7.2±0.4% [12]. Morita et al studied the effect of troglitazone treatment for dexamethasone-induced glucose intolerance in five healthy men, and showed that the 2-week troglitazone administration reduced the 3-day dexamethasone administration-induced increase of the mean area under the curve from 0 to 3 h for both plasma glucose and serum insulin concentrations during a 75-g oral glucose tolerance test [13]. These studies include premature factors such as monitor of diabetes [11], a short study period and small number of participants [11-13].

Recently, van Raalte et al performed a randomized, placebo-controlled, double-blind, crossover study in eight healthy men, to understand whether treatment with the GLP-1 agonist, exenatide could prevent glucocorticoids-induced glucose intolerance. Participants received three therapeutic regimens for two consecutive days: 1) 80 mg of oral prednisolone everyday and intravenous exenatide infusion; 2) 80 mg of oral prednisolone and intravenous saline infusion; and 3) oral placebo-prednisolone and intravenous saline infusion. Oral prednisolone and intravenous saline infusion increased postprandial glucose levels, which was prevented by the concomitant infusion of exenatide. Exenatide reduced prednisolone-induced hyperglucagonemia during the meal challenge. Oral prednisolone and intravenous saline infusion decreased the first-phase glucose- and arginine-stimulated C-peptide secretion, whereas oral prednisolone and intravenous exenatide infusion improved the first- and second-phase glucose- and arginine-stimulated C-peptide secretion, suggesting the effectiveness of the incretin-based therapy for the prevention of steroid diabetes [14]. However, this is the study to report acute effect of the incretin-based therapy for steroid-induced glucose intolerance in healthy individuals.

Jensen et al reported that glucocorticoid-induced glucose intolerance is associated with a progressive decline of incretin effects [19], suggesting the incretin-based therapy including the DPP-4 inhibitors may be useful for the treatment of glucocorticoid-induced diabetes. We previously reported a patient with glucocorticoid-induced diabetes whose glucose levels were ameliorated by the use of DPP-4 inhibitor, sitagliptin [20]. Ohashi et al reported that alogliptin improved steroid-induced hyperglycemia by decrease of glucagon levels through an increase in plasma GLP-1 levels, in 11 patients with chronic kidney disease. The mean ± SD of alogliptin treatment periods was 11.4 ± 12.8 days [21]. van Genugten et al studied effects of the co-administration of daily 100 mg sitagliptin with daily 30 mg prednisolone for 2 weeks in men with the metabolic syndrome. However, sitagliptin could not prevent prednisolone-induced increment in postprandial glucose concentrations [22]. To our knowledge, our study is the first to report chronic effect (6 months) of the DPP-4 inhibitor on glucose metabolism including HbA1c, in patients with long-standing steroid-induced diabetes. The present study also demonstrated a significant and inverse correlation between changes in HbA1c levels and HbA1c levels at baseline, suggesting that sitagliptin is more effective to improve glycemic control with glucocorticoid-treated patients with poorer glucose control.

Glucocorticoids usually induce weight gain [23]; however, our study showed that the sitagliptin treatment significantly reduced body weight by 2.7 kg. In our previous study, sitagliptin also significantly reduced body weight after the 6-month treatment in patients with type 2 diabetes, and a significant and negative correlation between change in body weight and body mass index at baseline was observed [24]. Sitagliptin may reduce body weight by ameliorating obesity-related factors such as insulin resistance. However, the underlying mechanism for sitagliptin-mediated reduction of body weight in patients taking glucocorticoids remains unknown, which should be elucidated in the future.

The present study has several limitations. First, other hypoglycemic agents, food intakes and/or exercise levels may have an influence on the study results. Second, we could not exclude the influence of diseases treated with glucocorticoids. Third, our analysis included both patients with glucocorticoid-induced diabetes and patients with pre-existing type 2 diabetes. The number of studied subjects was small. Further studies, preferably with larger numbers of subjects, will be needed.

In conclusion, we studied effects of 6-month sitagliptin treatment on metabolic parameters in diabetic patients taking oral glucocorticoids, and found that sitagliptin significantly reduced plasma glucose, HbA1c and body weight. In the present study, sitagliptin is more effective to improve glycemic control in diabetic patients taking glucocorticoids with higher HbA1c levels, independently of the daily glucocorticoid dose.
